# Extending the range of differential diagnosis of chronic traumatic encephalopathy of the boxer: Insights from a case report

**DOI:** 10.1590/1980-57642018dn12-010014

**Published:** 2018

**Authors:** Leonardo Caixeta, Iron Dangoni, Rafael Dias de Sousa, Pedro Paulo Dias Soares, Andreia Costa Rabelo Mendonça

**Affiliations:** 1 Associate Professor of Neurology, Federal University of Goiás, Goiânia, GO, Brazil. Coordinator, Cognitive and Behavioral Neurology Unit, Department of Neurology, Hospital das Clínicas; 2 Behavioral and Cognitive Neurology Unit, Department of Neurology, Hospital das Clínicas, Federal University of Goiás, Goiânia, GO, Brazil

**Keywords:** chronic traumatic encephalopathy, dementia pugilistica, differential diagnosis, frontal lobe dementia, corticobasal syndrome, multiple system atrophy, encefalopatia traumática crônica, demência pugilística, diagnósticos diferenciais, demência do lobo frontal, degeneração corticobasal, atrofia de múltiplos sistemas

## Abstract

Sports activities associated with repetitive cranial trauma have become a fad and are popular in gyms and even among children. It is important to consistently characterize the consequences of such sports activities in order to better advise society on the real risks to the central nervous system. We present the case of a former boxer reporting cognitive and behavioral symptoms that began six years after his retirement as a boxer, evolving progressively with parkinsonian and cerebellar features suggestive of probable chronic traumatic encephalopathy (CTE). Using our case as a paradigm, we extended the range of differential diagnosis of CTE, including corticobasal degeneration, multiple system atrophy, vitamin B12 deficiency, neurosyphilis, frontotemporal dementia and Alzheimer’s disease.

## RESUMO

As atividades esportivas associadas ao trauma craniano repetitivo tornaram-se uma moda e são populares nas academias e entre as crianças. É importante fazer uma caracterização consistente das consequências de tais atividades esportivas, a fim de aconselhar melhor uma sociedade sobre os riscos reais para o sistema nervoso central. Apresentamos um antigo boxeador relatando sintomas cognitivos e comportamentais que começaram seis anos após sua aposentadoria como boxeador e evoluiu progressivamente com características parkinsonianas e cerebelares sugestivas de provável encefalopatia traumática crônica (ETC). Usando nosso caso como paradigma, ampliamos a gama de diagnóstico diferencial de ETC, incluindo degeneração corticobasal, atrofia de múltiplos sistemas, deficiência de vitamina B12, neurossífilis, demência frontotemporal e doença de Alzheimer.

We live in a time when sports activities associated with repetitive cranial trauma (boxing, MMA) have become a fad. They are very popular in regular gyms and even among children.^1^^,^ 2 It is important for neuropsychiatry to consistently characterize the consequences of such sports activities in order to better advise society on the real risks to the central nervous system. Chronic traumatic encephalopathy (CTE) refers to a neurodegenerative disease related to the long-term consequences of repetitive cranial trauma.^3^ Some authors consider dementia pugilistica (punch-drunk syndrome, boxer’s dementia) the most serious and terminal stage of CTE,^4^ while others regard it as a condition that exists along a continuum: from mild neurocognitive deficits to full dementia.^5^ From a clinical standpoint, boxers presenting CTE will exhibit varied combinations of motor, cognitive and behavioral alterations.^6^ The diagnosis of CTE depends on documenting progressive encephalopathy that is consistent with the clinical symptoms of TBI explained by brain trauma rather than being attributable to an alternative pathophysiological process.^3^

The CTE associated with boxing has a prevalence of about 20% among professional boxers. Risk factors classically related to CTE include the frequency of repeated exposure (i.e., career length, total number of fights, retirement age), technical supervision intensity, sparring increase, and apolipoprotein genotype (APOE).^7^ Dementia pugilistica has been included among the acquired tauopathies, but the pathophysiology of CTE is still under scrutiny, although it has been suggested that neuroinflammation can drive the association between tau hyperphosphorylation and dementia development following traumatic brain injury.^8^

Chronic traumatic encephalopathy has been associated with some forms of dementia, both clinically and neuropathologically.^9^ Clinically, there may be several common elements between dementia pugilistica and Alzheimer’s disease,^10^ for example. On the other hand, the relationship and common aspects between CTE and other rarer forms of dementia have not been the subject of much discussion. We present a case of CTE that provides a differential diagnosis with other unusual forms of dementia.

## CASE REPORT

We describe the case of Mr. G, a 79-year-old Brazilian male, brown skin, divorced, former boxer, retired, with nine years of education.

The patient fought as a boxer for 16 years (from 17 to 33 years old), in the light middleweight category. He had about 14 official fights. Two knockdowns were recorded and he had a severe nose fracture, having spent two years without a fight.

In 1977 (he was then 39 years old), approximately six years after retirement as a boxer (at the time working as a real-estate broker), he started taking his wife along as a companion to visit customers in order to avoid mistakes in finding addresses, due to topographical disorientation. The marital relationship, initially healthy, was ruined by the patient’s pathological jealousy (Othello’s syndrome).

In 2000, when cognitive deterioration was noticed, Mr. G presented a picture of anomie for his children’s names, managing to remember by speaking the names of all of them, from the oldest to the youngest, until he reached the desired name. Memory impairment also extended to bank passwords, phone numbers, recent events and even old ones.

From 2003, with the progressive worsening of the clinical presentation, he forgot the milk on the burner and sometimes even the cap on his head, besides beginning to experience difficulties managing his accounts. The patient become very upset by the memory lapses, especially when he could not remember politicians’ names, content of reports or his religious prayers.

From 2013, the patient reported tremors, sensations of heaviness and numbness in his legs, besides imbalance, which begun to affect his gait, having suffered several falls in the previous three years.

In 2015, he was diagnosed with depression, presenting symptoms of depressive mood, propensity to cry, anxiety, insomnia, fatigability and dysphoria. He was prescribed Fluoxetine at the time, which we later switched to Quetiapine. Currently, the patient reports social isolation, mainly due to forgetfulness and slow thinking, complaining that he sometimes does not know what to say to friends. He often misses appointments, and has to write down tasks on his hand so as not to forget, and needs help with medication. He was diagnosed with neurogenic bladder syndrome and, in recent years, presented with sexual impotence. Mr. G has no history of alcoholism and has high blood pressure. There are no reports of dementia diagnosed in the family. The patient is in use of losartan 50 mg/day and atenolol 50 mg/day.

On the neurological examination, he presented parkinsonian changes indicated by gait problems with absence of bilateral arm swinging, difficulty getting up from a chair without support, exhibited postural instability, including a positive pull test, associated with plastic hypertonia (cogwheel) and light atypical tremor, both on the right side, bradykinesia and hypomimia. On the evaluation of coordination and cerebellar functions, dysmetria was observed on the index-nose test (worse with eyes closed), as well as positive Romberg when sensitized ( eyes closed). He presented with visible primitive reflexes and exacerbated tendon reflexes, as well as proprioceptive impairment.

On the psychic examination, the patient presented signs of frontal disinhibition, characterized by logorrhea, evident pressure of speech, long-winded speech, with several interruptions of the interlocutor and lack of insight. The patient showed suspicion (paranoid thoughts) to the point of threatening not to return to the consultation if there was insistence of the presence of a companion (at the request of the attending physician). The patient’s irritability or aggressiveness during the physic examination was not evident, but there was a previous report of dysphoria, nervousness and anxiety, as well as marital problems.

**Figure 1:**
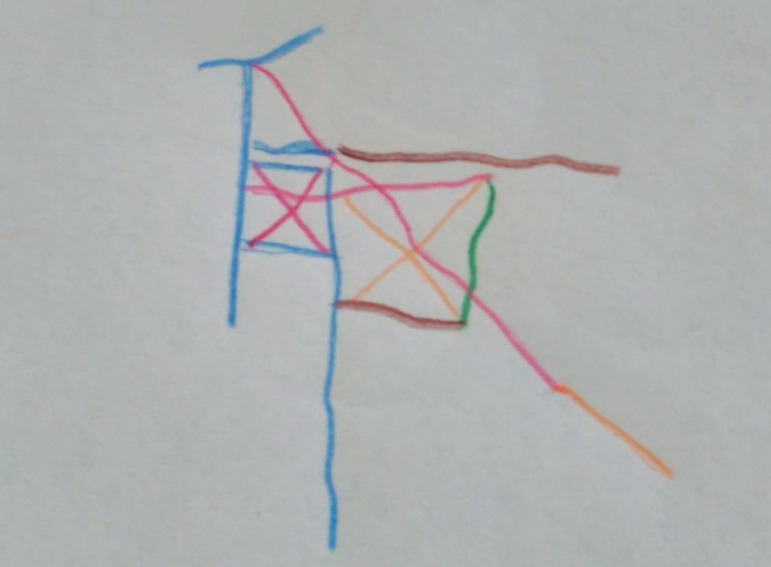
Patient’s copy of Rey Complex Figure showing lack of planning, perseveration, visuoconstructional and visuomotor difficulties.

On the cognitive examination, he scored 23 points on the Mini-Mental State Examination, dropping 4 points on calculation, two on recall and one on the figure copy. He had evident dyscalculia. On the *Boston Naming Test*, he named only one item out of 15. In writing, he showed omissions and letter switching, phrases with isolated words without links and paragraph. On the *Boston Cookie Theft*, he noted only the details, but could not produce a ‘gestalt’ of the figure. He successfully performed the verbal fluency test (animals’ category) (14 animal names). On the *Stroop Test*, performance was 7SDs below on the first two cards and 2SDs below on the third card, with 16 errors, demonstrating impulsivity and lack of inhibitory control. On *Rey’s Complex Figure* (Figure 1), he was unable to copy it within the five minutes available. He presented difficulty in mental control (inability to perform months backwards and also the serial sevens).

On the *Trail Making Test A*, he exhibited slowed information processing and on the *Trail Making Test B*, lack of strategy and flexibility of thought, also showing working memory impairment (requiring several repetitions of the rule). Despite this, he could not complete the test in 5 minutes and made many mistakes. In the labyrinths, the same process occurred, with several perseverations. On the *RAVLT*, memory had a lower-than-expected acquisition curve, with major difficulty after intrusion and impaired delayed recall (2SDs). He did not benefit from clues, scoring 2SDs below the expected score for his age, showing several intrusions and perseverations in the attempts of the acquisition curve, as well as on the delayed recall and recognition.

**Figure 2:**
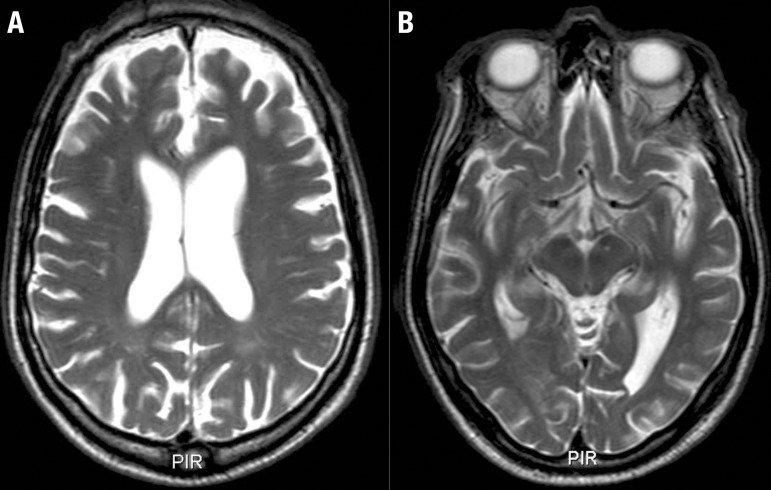
Brain MRI (T2-weighted axial sections) revealed: [A] moderate pre-frontal atrophy, especially in the medial and dorsolateral areas and asymmetric ventricular dilatation (greater in the left hemisphere); [B] asymmetric atrophy (greater in the left hemisphere).

**Figure 3:**
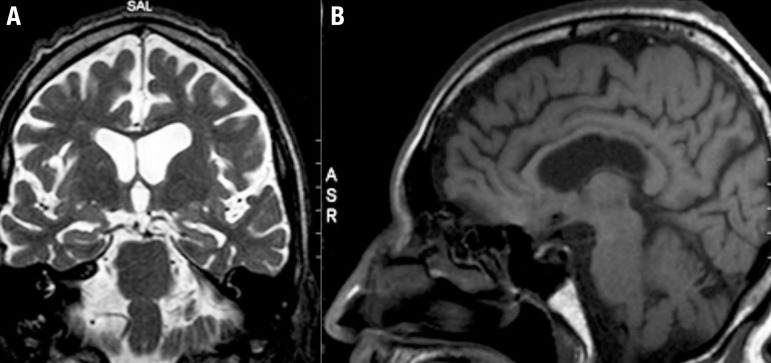
Brain MRI showing: [A] (T2-weighted coronal section) mild hippocampal atrophy; [B] (sagittal T1 section) demonstrates: mild cerebellar and posterior callosum atrophy.

Initially, the differential diagnoses was B12 deficiency and Tabes Dorsalis, which supposedly explained the changes in gait and postural balance, proprioceptive changes and some of the cognitive and behavioral symptoms. The patient was submitted to the protocol of exams for evaluation of cognitive disorders (blood count, urine test, renal and hepatic function, serum glucose, TSH, free T4, folic acid, vitamin B12, VDRL and FTA-abs, anti-HIV), all tested normal, in view of which the aforementioned hypothesis was rejected. Findings on brain MRI included focal structural alterations, disproportionate for the patient’s age and therefore suggestive of a degenerative process (shown in Figures 2 and 3).

The use of atypical antipsychotics for the control of delusional jealousy was attempted, but the patient had no adherence to any of the proposed treatments.

## DISCUSSION

Our case meets diagnostic criteria for CTE.^3^ The neuropsychological profile of this CTE case is complex and heterogeneous, with impairments in various cognitive domains (executive functions and attention, naming, coding, recovery, praxis, visuospatial functions, and processing speed), suggesting both cortical and subcortical impairment in anterior and posterior brain areas, medial and lateral, in both left and right hemispheres. Other reviews on this theme also found a wide range of neuropsychological changes associated with boxing,^4^ thus, there is no pathognomonic cognitive profile of CTA.

The clinical picture of chronic traumatic encephalopathy is also proteiform; therefore, in our case a broad range of differential diagnoses can be entertained:

*1) Corticobasal degeneration (CBD) -* The clinical symptomatology of our case resembles, in part, features of a corticobasal syndrome: asymmetric parkinsonism (hypertonia and tremor in the right side only), asymmetric dyspraxia, bradykinesia, hypomimia, and gait disturbance.^11^ Neuropsychological findings indicating fronto-subcortical impairment represented by executive dysfunction, anomia, and apraxia also fit the cognitive profile of CBD. Moreover, neuroimaging also corroborates this impression (asymmetric atrophy, most evident on the left side and frontal atrophy - see Figure 1). Some authors classify CBD as part of the frontotemporal lobar degeneration group^12^ and thus can present with behavioral changes. In fact, our patient has a disinhibition syndrome characterized by excessive verbal output, pressure of speech and lack of insight. Although this is the most important differential diagnosis in our case, the presence of cerebellar symptoms makes this possibility less likely.

*2) Frontotemporal dementia (FTD) -* As in CTE (and similar to our case), symptoms usually emerge in the presenium, behavior and personality changes are early, always present and important in the clinical scenario; neuropsychological deficits are mainly related to executive dysfunction and impairment of fronto-subcortical circuits. In addition, the MRI in our case showed focal frontal atrophy. FTD eventually can present with parkinsonian symptoms and other features of subcortical pathology,^13^ which may produce additional similarity between pugilistic dementia and FTD. In fact, our case has some of the clinical characteristics of FTD, but lacks core clinical features and does not meet diagnostic criteria for FTD. Other authors have addressed the similarities between FTD and CTE.^14^

*3) Multiple System Atrophy (MSA) -* Also a proteiform disease with many features that resemble CTE: a mixture of extrapyramidal, cerebellar, behavior and sometimes, pyramidal symptoms, causing dementia only in its final stages.^15^ Pugilistic dementia can, therefore, from a certain perspective, be considered a disease that affects multiple systems, akin to MSA. Interestingly, Koga^16^ found, in a brain bank of MSA carriers, some cases with pathology compatible with CTE, perhaps related to a greater number of falls (and consequently head injury) experienced by MSA patients or possibly because, according to our hypothesis, MSA can actually constitute one of the possible phenotypes of CTE. Our patient also presented some symptoms of dysautonomy (sexual impotence, neurogenic bladder), besides cerebellar and extrapyramidal features. In spite of the similarities, our patient presented no additional symptoms of dysautonomy and no marked cerebellar atrophy, as occurs in MSA, and thus he does not meet diagnostic criteria for MSA.^15^

*4) Neurosyphilis -* Traditionally known as “the great imitator”, also a disease that can manifest with various different combinations of motor, cognitive and behavioral symptoms. It is considered an important differential diagnosis in countries where syphilis is still endemic.^17^ Although our case presents some symptoms that could suggest neurosyphilis (proprioceptive and gait abnormalities, alteration of reflexes, maniform behavior), this diagnosis was ruled out by normal VDRL.

*5) B12 deficiency -* Vitamin B12 deficiency may manifest with sensory symptoms associated with myelopathy (altered proprioceptive sensitivity and consequent impaired static and dynamic balance with altered tendon reflexes), as well as cognitive symptoms, mood disturbance, psychosis, and even extrapyramidal and other motor symptoms.^18^ Although our case has some symptoms that might suggest B12 deficiency (cognitive changes, symptoms of depression, proprioceptive and gait changes, and reflex changes), this diagnosis was ruled out by normal vitamin B12.

*6) Alzheimer’s Disease -* Although our patient has memory complaints, episodic memory does not seem to be the dominant element in the reported case, as occurs in Alzheimer’s disease. Moreover, temporal mesial atrophy (mild hippocampal atrophy) present on the patient’s MRI is also described in cases of Traumatic Encephalopathy. In other words, it is not exclusive to Alzheimer’s, but represents an important differential diagnosis in the case. The diagnosis of Alzheimer’s disease is unlikely since the patient exhibits changes that are not usually involved in this pathology, such as parkinsonism, subcortical and cerebellar manifestations. On the one hand, there are alterations that are not features of Alzheimer’s disease. However, the case lacks typical manifestations, for example, significant episodic memory loss. The patient’s deficit is not as pronounced as in Alzheimer´s disease.

As a limitation of our study, we should note that the causal nexus between our patient’s current dementia and his background as a boxer several decades earlier cannot be proven conclusively. Our case does not have anatomopathological data. Nevertheless, our case fulfills clinical criteria for pugilistic dementia and we cannot identify another form of dementia that could better explain the proteiform symptoms, as well as the structural alterations presented.

Sports with repetitive cranial trauma such as boxing have received increasing attention and acceptance from the public in recent years.^1^ We suggest that our case provides additional evidence that boxing may represent a risk factor for subsequent onset of cognitive and behavioral alterations, putting at risk the future generations that practice this sport modality, even as amateurs.
